# Value of peak flow rates measured during a spontaneous breathing trial to predict success of weaning from mechanical ventilation

**DOI:** 10.1186/cc13487

**Published:** 2014-03-17

**Authors:** K Gundogan, S Baldane, R Coskun, I Bahar, G Altunyurt, H Mumcuoglu, M Guven, M Sungur

**Affiliations:** 1 Erciyes University, Kayseri, Turkey

## Introduction

Numerous parameters have been suggested for the prediction of weaning from mechanical ventilation; however, these parameters have limited success in the prediction of weaning outcome. The aim of this study is to assess the success of minute peak flow rates (spontaneous peak inspiratory flow rate (SPIF) and spontaneous peak expiratory flow rate (SPEF)) measured during a spontaneous breathing trial (SBT) in the prediction of weaning outcome.

## Methods

Patients managed and receiving mechanical ventilation support for at least 24 hours in the medical and surgical ICUs of Erciyes University between March 2011 and May 2012 were included in the present study. Over 30 minutes, SPIF and SPEF values were measured during a SBT in patients spontaneously breathing by T tube. Patients who tolerated 30 minutes of SBT were extubated. Patients who did not need reintubation for 48 hours after extubation were considered successful weaning, while those needing reintubation were considered weaning failure.

## Results

The study was completed with 36 patients overall, being 11 patients in failure and 25 patients in the success group. In both groups, areas under the curve (AUCs) were calculated for each minute via ROC analysis using minute SPIF and SPEF values measured during SBT. The maximum AUC was calculated at minute 23 for SPIF (0.564; 95% CI: 0.363 to 0.764) and at minute 9 for SPEF (0.542; 95% CI: 0.316 to 0.376). Cutoff values were determined for the minutes in which maximum AUC values for SPIF and SPEF were detected; and sensitivity and specificity values were calculated. When the cutoff value for SPIF was accepted as >26.7 l/minute at minute 23, sensitivity and specificity was calculated as 72.0% and 28.0%, respectively. When the cutoff value for SPEF was accepted as >24.7 l/minute at minute 9, sensitivity and specificity were calculated as 63.6% and 48.8%, respectively.

## Conclusion

We think that minute SPIF measurement which has better sensitivity and minute SPEF measurement which has better specificity compared with available traditional predictors [1] may be used as a potential bedside weaning predictor when evaluated in comprehensive studies.
